# Heterogeneity and complexity within the nuclease module of the Ccr4-Not complex

**DOI:** 10.3389/fgene.2013.00296

**Published:** 2013-12-23

**Authors:** G. Sebastiaan Winkler, Dario L. Balacco

**Affiliations:** School of Pharmacy, Centre for Biomolecular Sciences, University of Nottingham, University ParkNottingham, UK

**Keywords:** deadenylase, mRNA turnover, poly(A), ribonuclease, Pop2, Calif

## Abstract

The shortening of the poly(A) tail of cytoplasmic mRNA (deadenylation) is a pivotal step in the regulation of gene expression in eukaryotic cells. Deadenylation impacts on both regulated mRNA decay as well as the rate of mRNA translation. An important enzyme complex involved in poly(A) shortening is the Ccr4-Not deadenylase. In addition to at least six non-catalytic subunits, it contains two distinct subunits with ribonuclease activity: a Caf1 subunit, characterized by a DEDD (Asp-Glu-Asp-Asp) domain, and a Ccr4 component containing an endonuclease-exonuclease-phosphatase (EEP) domain. In vertebrate cells, the complexity of the complex is further increased by the presence of paralogs of the Caf1 subunit (encoded by either *CNOT7* or *CNOT8*) and the occurrence of two Ccr4 paralogs (encoded by *CNOT6* or *CNOT6L*). In plants, there are also multiple Caf1 and Ccr4 paralogs. Thus, the composition of the Ccr4-Not complex is heterogeneous. The potential differences in the intrinsic enzymatic activities of the paralogs will be discussed. In addition, the potential redundancy, cooperation, and/or the extent of unique roles for the deadenylase subunits of the Ccr4-Not complex will be reviewed. Finally, novel approaches to study the catalytic roles of the Caf1 and Ccr4 subunits will be discussed.

## INTRODUCTION

Virtually all mature protein-coding mRNAs in eukaryotic cells contain a 5′ cap structure and a 3′ poly(A) tail, with the notable exception of mRNAs encoding histones. Both modifications play a critical role in translation and mRNA turnover. In mRNA turnover, shortening of the poly(A) tail (deadenylation) is the initial and often rate-limiting step (Parker and Song, 2004; Goldstrohm and Wickens, 2008; Wahle and Winkler, 2013). Following deadenylation, recruitment of the decapping enzyme complex is followed by exonucleolytic degradation from the 5′ end by Xrn1, or by the exosome complex with 3′-5′ polarity (Parker and Song, 2004; Garneau et al., 2007; Houseley and Tollervey, 2009).

Deadenylation may also influence protein synthesis. During translation, the efficiency of initiation is enhanced by interactions between the cap and the poly(A) tail, which are mediated by the poly(A)-binding protein (PABP), the cap-binding factor eIF4E, and the intermediary scaffold eIF4G (Munroe and Jacobson, 1990; Gallie, 1991; Wells et al., 1998). Consistent with the notion that the poly(A) tail contributes to initiation of translation is the observation that poly(A) tail length correlates with ribosome binding, a measure for translational efficiency, in *Schizosaccharomyces pombe* (Lackner et al., 2007).

Several enzyme complexes are implicated in deadenylation. These include the conserved, trimeric Pan2-Pan3 complex, composed of a single Pan2 catalytic subunit bound to a Pan3 dimer, the PARN deadenylase, which is absent in single cellular eukaryotes, and less-well characterized enzymes, such as the circadian deadenylase Nocturnin, and the Caf1z-Ccr4d complex (reviewed in Godwin et al., 2013; Wahle and Winkler, 2013). However, in all model systems examined (*Saccharomyces cerevisiae*, *Caenorhabditis elegans*, *Drosophila melanogaster*, and human cells), the multisubunit Ccr4-Not complex has been identified as the main deadenylase (Tucker et al., 2001; Temme et al., 2004; Yamashita et al., 2005; Nousch et al., 2013).

The activity of the deadenylase enzymes appears to be targeted to specific mRNAs by RNA-binding proteins. The following paradigms have been established for recruitment of the Ccr4-Not deadenylase: targeting mediated by direct interactions between Ccr4-Not and the RNA-binding protein recognizing a linear RNA sequence, as exemplified by pumilio proteins, or the ARE-binding protein tristetraprolin (TTP; Goldstrohm et al., 2006; Sandler et al., 2011); by RNA-binding partners recognizing structural elements, such as an RNA stem loop structure (Roquin; Leppek et al., 2013); via ternary complexes, as indicated by recruitment involving the RNA-binding protein CPEB3, which is mediated via Tob1, thus forming a ternary CPEB3/Tob1/Ccr4-Not complex (Hosoda et al., 2011). Finally, an important mechanism for the recruitment of both Ccr4-Not and the Pan2-Pan3 complex to specific mRNAs involves interactions with GW182/TNRC6 proteins, which are part of the miRNA repression complex (Chekulaeva et al., 2011; Fabian et al., 2011; Kuzuoglu-Ozturk et al., 2012; Huntzinger et al., 2013). In addition to these mechanisms of recruitment, the Pan2-Pan3 and Ccr4-Not deadenylases can also bind to the poly(A)-binding protein (PABP). In the case of the Pan2-Pan3 complex, this is mediated by the presence of a short PAM2 motif in the Pan3 protein, which can interact directly with the PABP C-terminal domain (Siddiqui et al., 2007). By contrast, none of the subunits of the Ccr4-Not complex contain a PAM2 motif. However, this motif is present in both the Tob1 and Tob2 protein, which directly interact with the Ccr4-Not complex (Ezzeddine et al., 2007). Thus, where the Pan2-Pan3 complex can interact directly with the PABP, this interaction is indirect in case of the Ccr4-Not complex.

## OVERVIEW OF THE Ccr4-Not COMPLEX

The Ccr4-Not complex contains two components that are associated with deadenylase activity: the Ccr4 and Caf1 subunits (**Table 1**). Carbon catabolite repression (ccr) 4 was originally identified as a regulator of alcohol dehydrogenase II in the yeast *Saccharomyces cerevisiae* (Denis, 1984). Likewise, Ccr4-associated factor (Caf) 1 was originally isolated in yeast as PGK promoter directed over production (pop) 2, a mutant overproducing mouse α-amylase under control of a PGK promoter (Sakai et al., 1992; Draper et al., 1995). In addition to its nuclease subunits, several non-catalytic components have been identified (reviewed in Collart and Panasenko, 2012; Wahle and Winkler, 2013). Most of the genes encoding these subunits, including *NOT1*, *NOT2*, *NOT3* as well as *NOT4*, were first identified in yeast using a genetic screen for transcriptional regulators (Collart and Struhl, 1994).

**Table 1:** Standard names and synonyms of the Ccr4-Not nuclease components.

	Caf1		Ccr4	
	Gene	Synonyms	Gene	Synonyms
*Saccharomyces cerevisiae*	POP2	CAF1	CCR4	FUN27, NUT21
*Schizosaccharomyces pombe*	caf1	pop2	CCR4	
*Caenorhabditis elegans*	CCF-1		CCR-4	
*Drosophila melanogaster*	Pop2	CAF1	Twin	CCR4
*Xenopus laevis*	Cnot7	caf1, caf-1	Cnot6	CCR4A
	Cnot8	pop2, calif	Cnot6l	CCR4B
*Danio rerio*	Cnot7	zgc:153168	Cnot6	Zgc:65822
	Cnot8	zgc:63844	Cnot6l	Zgc:111987
*Mus musculus*	Cnot7	Caf1	Cnot6	
	Cnot8		Cnot6l	
*Homo sapiens*	CNOT7	Caf1; Caf1a	CNOT6	Ccr4; Ccr4a
	CNOT8	Pop2; Calif; Caf1b	CNOT6L	Ccr4-like;

In *Saccharomyces cerevisiae*, two variant complexes with a distinct molecular weight of 1.0 and 1.9 MDa are present (Liu et al., 1997; Liu et al., 1998; Chen et al., 2001). The overall structure of the yeast 1.0 MDa complex is L-shaped with the Caf1 and Ccr4 components located in the hinge connecting the two arms (Nasertorabi et al., 2011). In *Homo sapiens*, the Ccr4-Not complex has an estimated molecular weight of 1.2 MDa. Importantly, while the Not4 subunit appears to be an integral component of the yeast complex, CNOT4, its human ortholog, resides in a separate complex with an estimated molecular weight of 200 kDa (Lau et al., 2009).

## THE Ccr4 SUBUNIT, ORTHOLOGS, AND PARALOGS

**FIGURE 1:**
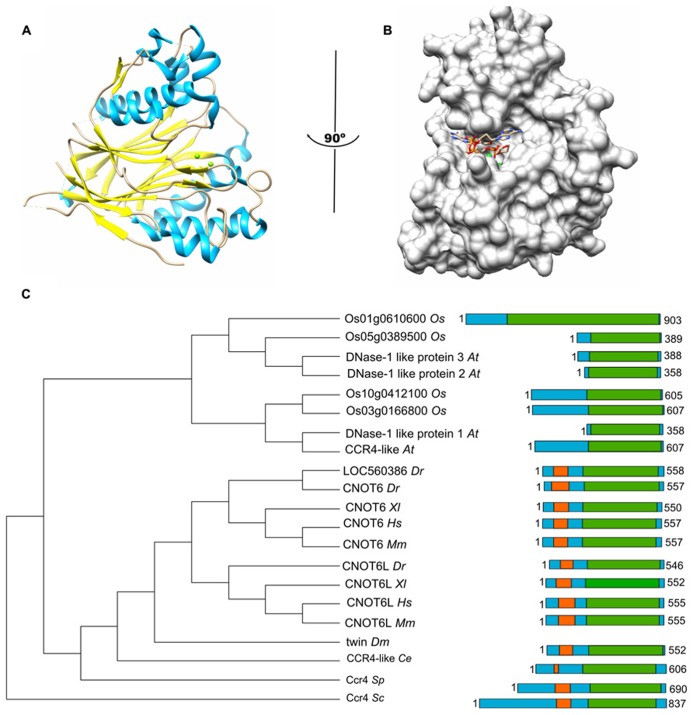
**The Ccr4 nuclease subunit.** **(A)** Structure of the human Ccr4/CNOT6L catalytic domain. The nuclease domain forms an α/β sandwich typical for the endonuclease-exonuclease-phosphatase (EEP) domain. The Mg(II) ions located in the active site are indicated in green; α-helical regions, blue; β-strands, yellow. **(B)** Binding of poly(A) by human Ccr4/CNOT6L. The Mg(II) ions located in the active site are indicated in green. Structures (using PDB accession number 3NGO) were visualized using the UCSF Chimera package (http://www.cgl.ucsf.edu/chimera; Pettersen et al., 2004). **(C)** Evolutionary conservation of Ccr4 across the eukaryotic kingdom. Sequence analysis was carried out using Basic local alignment search tool (BLAST) in combination with the Reference protein database. Only homologs with >75% sequence coverage as compared to *Saccharomyces cerevisiae* (*Sc*) Ccr4 were selected from the following species: *Schizosaccharomyces pombe* (*Sp*), *Caenorhabditis elegans* (*Ce*), *Drosophila melanogaster* (*Dm*), *Danio rerio* (*Dr*), *Xenopus laevis* (*Xl*), *Mus musculus* (*Mm*), *Homo sapiens* (*Hs*), *Arabidopsis thaliana (At)*, and *Oryza sativa (Os)*. The EEP domain is indicated in green; the leucine-rich repeat domain is highlighted in orange.

The Ccr4 subunit is characterized by the presence of two domains: an amino-terminal leucine-rich repeat (LRR) domain, and a carboxy-terminal endonuclease-exonuclease-phosphatase (EEP) domain. The latter is associated with its ribonuclease activity. Analysis of the enzymatic activity of human and yeast Ccr4 indicates that the enzyme has a strong preference for poly(A) residues *in vitro* (Chen et al., 2002; Wang et al., 2010). A crystal structure of the nuclease domain indicates that the C-terminus forms an α/β-sandwich fold, which is very similar to that of other hydrolyses such as apurinic/apyrimidinic endonuclease (APE) 1 (**Figure 1A**; Wang et al., 2010). Two Mg(II) ions are required for hydrolysis of the phosphoester backbone. The metal ions are coordinated by an asparagine, glutamate, two aspartate, and a histidine residue, which are part of short sequence motifs that are highly conserved in the ExoIII/APE1 family of DNA and RNA nucleases. Substitutions of amino acids involved in the coordination of the metal ions abolish the enzymatic activity (Chen et al., 2002; Wang et al., 2010). The Mg(II) ions are located at the bottom of a narrow cleft in which the nucleic acid substrate is inserted (**Figure 1B**). The crystal structure of the nuclease domain in complex with an oligo(A) DNA molecule highlights some features of the selective interaction with poly(A), which involve a specific interaction between a backbone carbonyl and the 6′ amino group of the adenosine base (Wang et al., 2010).

In addition to the nuclease domain, Ccr4 contains an amino-terminal LRR domain (Malvar et al., 1992; Dupressoir et al., 2001). The LRR repeats are composed of alternating α-helices and β-sheets, which form a moderately curved solenoid structure with the β-sheets located on the concave side, and the α-helices exposed on the convex curvature (Basquin et al., 2012). The LRR domain provides an interaction surface with the Caf1 subunit (Dupressoir et al., 2001; Clark et al., 2004).

In the single cellular yeasts *Saccharomyces cerevisiae*, *Schizosaccharomyces pombe*, as well as the metazoans *Drosophila melanogaster* and *Caenorhabditis elegans*, a single Ccr4 subunit is present. By contrast, two paralogs are present in vertebrates, including *Danio rerio*, *Xenopus laevis*, *Mus musculus*, and *Homo sapiens* (**Figure 1C**; Dupressoir et al., 2001; Morita et al., 2007; Cooke et al., 2010). Both Ccr4 paralogs (encoded by *CNOT6* or *CNOT6L*) can associate with the Ccr4-Not complex, but the two paralogs cannot co-exist in the same complex (see below for further details). In addition, inspection of the non-redundant protein databank using the Basic local alignment search tool (BLAST) identified several homologs of yeast Ccr4 in *Oryza sativa* and *Arabidopsis thaliana* >75% sequence coverage as compared to *Saccharomyces cerevisiae* Ccr4. However, none of these plant homologs contain the characteristic amino-terminal LRR domain. Indeed, these proteins appear to be more related to eukaryotic Ccr4 homologs that are not associated with the Ccr4-Not complex. For instance, *Saccharomyces cerevisiae* contains three non-essential homologs of Ccr4 (Ngl1, Ngl2, and Ngl3). While Ngl2 is involved in the processing of the 5.8S ribosomal precursor RNA (Faber et al., 2002), Ngl3 is proposed to play a role in cellular deadenylation independent of Ccr4-Not (Feddersen et al., 2012). Several human Ccr4 homologs lacking an LRR domain (Nocturnin/NOC/CCRN4L, Angel, Angel2, and PDE12) are also implicated in deadenylation (Godwin et al., 2013). Nocturnin is a circadian deadenylase that may associate with subunits of the Ccr4-Not complex in the absence of an LRR (Baggs and Green, 2003). Moreover, Angel homolog 2 (Ccr4d) binds to a distant homolog of Caf1, Caf1z/TOE1 (Wagner et al., 2007). This complex has deadenylase activity, but its biological function is yet unclear. Thus, the fact that the plant homologs do not contain the LRR domain, which is a characteristic for the yeast and vertebrate Ccr4 subunits, may suggest that plant Ccr4-Not may have a fundamentally different architecture.

## THE Caf1 SUBUNIT, ORTHOLOGS, AND PARALOGS

**FIGURE 2:**
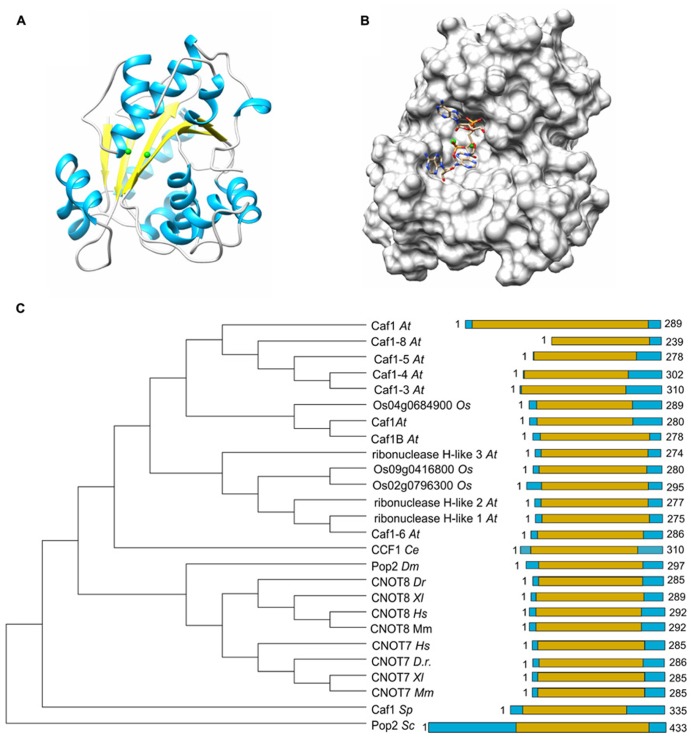
**The Caf1 nuclease component.** **(A)** Structure of the human Caf1/CNOT7 catalytic domain (PDB accession number 4GMJ). The Mg(II) ions located in the active site are indicated in green; α-helical regions, blue; β-strands, yellow. **(B)** Model of poly(A) binding by human Caf1/CNOT7. The model was derived from superposition the structure of the PARN deadenyase in complex with RNA (PDB accession number 2A1R). The Mg(II) ions located in the active site are indicated in green. Structures were visualized using the UCSF Chimera package (http://www.cgl.ucsf.edu/chimera; Pettersen et al., 2004). **(C)** Evolutionary conservation of Caf1 across the eukaryotic kingdom. Sequence analysis was carried out using Basic local alignment search tool (BLAST) in combination with the Reference protein database. Only homologs with >75% sequence coverage as compared to *Saccharomyces cerevisiae* (*Sc*) Caf1 were selected from the following species: *Schizosaccharomyces pombe* (*Sp*), *Caenorhabditis elegans* (*Ce*), *Drosophila melanogaster* (*Dm*), *Danio rerio* (*Dr*), *Xenopus laevis* (*Xl*), *Mus musculus* (*Mm*), *Homo sapiens* (*Hs*), *Arabidopsis thaliana (At)*, and *Oryza sativa* (*Os*). The DEDD domain is highlighted in yellow.

The Caf1 subunit is characterized by the presence of an RNAse D domain, which belongs to the DEDD (Asp-Glu-Asp-Asp) superfamily of proteins associated with ribonuclease (RNAse) and deoxyribonuclease (DNAse) activity. Crystal structures from *Saccharomyces cerevisiae*, *Schizosaccharomyces pombe*, and *H. sapiens* Caf1 proteins indicate a central core composed of β-sheets surrounded by α-helices (**Figure 2A**; Thore et al., 2003; Jonstrup et al., 2007; Horiuchi et al., 2009; Petit et al., 2012). As is the case for the catalytic EEP domain of the Ccr4 subunit, the Caf1 protein contains two Mg(II) ions, which are required for its enzymatic activity. The metal ions are coordinated by a single glutamate and three aspartate residues, and substitution of any of these amino acids abrogates the enzymatic activity (Thore et al., 2003; Jonstrup et al., 2007; Horiuchi et al., 2009). Other bivalent cations may also bind in the active site and modulate enzyme activity (Andersen et al., 2009). The *Saccharomyces cerevisiae* Caf1 protein is unusual as compared to its homologs in other species, because it contains a long amino-terminal extension. In addition, the metal-binding region contains a non-canonical sequence. Although the purified yeast protein displays ribonuclease activity, it has a broad specificity and no preference for poly(A) (Thore et al., 2003). Moreover, mutations of active site residues do not cause phenotypes in yeast (Viswanathan et al., 2004). Thus, the importance of the enzymatic activity of the *Saccharomyces cerevisiae* Caf1 protein is not unambiguous. By contrast, the *Schizosaccharomyces pombe* and *H. sapiens* Caf1 orthologs contain highly conserved active site residues and the ribonuclease activity has a preference for poly(A) residues (Bianchin et al., 2005; Jonstrup et al., 2007; Horiuchi et al., 2009). Moreover, mutation of a residue involved in Mg(II) coordination results in sensitivity to hydroxyurea of *Schizosaccharomyces pombe* (Takahashi et al., 2007). The activity of Caf1 is distributive and AMP is released as the reaction product (Bianchin et al., 2005).

The poly(A) binding site of Caf1 has a different shape and is significantly wider as compared to the substrate binding pocket of Ccr4 (**Figure 2B**). No structures are available of Caf1-RNA complexes, but a model for RNA recognition was proposed based on the superposition of the DEDD domains of Caf1 and PARN in complex with poly(A) (Wu et al., 2005; Jonstrup et al., 2007; Andersen et al., 2009). This led to the identification of conserved serine and leucine residues for selectivity and processivity, respectively (Andersen et al., 2009).

As is the case for Ccr4, Caf1 is conserved with a single subunit present in *Saccharomyces cerevisiae*, *Schizosaccharomyces pombe*, *Drosophila melanogaster*, and *C. elegans.* By contrast, two closely related paralogs (encoded by *CNOT7* and *CNOT8*) are present in the vertebrates *Danio rerio*, *Xenopus laevis*, and *Mus musculus* as well as *Homo sapiens* (**Figure 2C**). While both Caf1 paralogs are subunits of Ccr4-Not, their presence in the complex is mutually exclusive (see below for further details). In plants, there are multiple homologs of Caf1, which are distantly related to their counterparts in the fruit fly and vertebrates. Interestingly, the human Caf1z/TOE1, which is part of a complex with the distant Angel2/Ccr4d homolog that lacks an LRR domain, is more closely related to plant Caf1 homologs (Wagner et al., 2007). This may suggest that the plant Caf1 homologs form complexes with their respective Ccr4 partners via interactions that do not involve an LRR domain. It may be speculated that some of these complexes may contain the plant orthologs of the non-catalytic Ccr4-Not subunits.

## THE NON-CATALYTIC SUBUNITS

**FIGURE 3:**
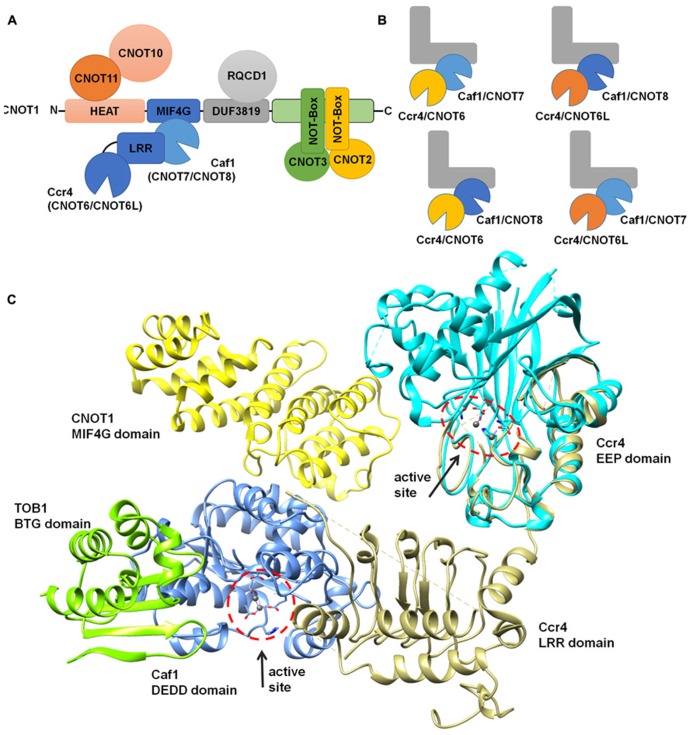
**Modular architecture of the Ccr4-Not complex.** **(A)** Schematic overview of the modular architecture of the mammalian Ccr4-Not complex. **(B)** Heterogeneity of the Ccr4-Not complex in vertebrate cells. The duplication of the catalytic subunits Caf1 (light blue, CNOT7; dark blue, CNOT8) and Ccr4 (yellow, CNOT6; orange, CNOT6L) may result in the formation of four highly related complexes. The non-catalytic components are represented in a L-shaped form (gray). **(C)** Structural overview of the nuclease sub-complex. Indicated are the MIF4G domain of human CNOT1 (yellow), the BTG domain of TOB1 (green), Caf1/CNOT7 (blue), yeast Ccr4 (khaki), and the catalytic EEP domain of human Ccr4/CNOT6L (cyan). The location and orientation of the active sites of Caf1 and Ccr4 are indicated and Mg(II) ions are shown (dark gray). The model was generated by superimposing the Tob-Caf1 structure (PDB accession number 2D5R), the MIF4G domain of human CNOT1 in complex with Caf1/CNOT7 (PDB accession number 4GMJ), and the EEP nuclease domain of human Ccr4/CNOT6L (PDB accession number 3NGO) on the structure of the yeast Not1-Caf1-Ccr4 complex (PDB accession number 4B8C). Molecular graphics and analyses were performed with the UCSF Chimera package (http://www.cgl.ucsf.edu/chimera; Pettersen et al., 2004).

The large Not1 (CNOT1) subunit is the central platform of the complex, on to which several modules are attached (Bai et al., 1999). The nuclease sub-complex is anchored to the central MIF4G domain, which is composed of several α-helices and which binds to the Caf1 subunit (**Figures 3A,C**; Bai et al., 1999; Basquin et al., 2012; Petit et al., 2012). Interactions between Caf1 and the LRR domain of Ccr4 are essential for stable interaction of the Ccr4 subunit to the complex (Draper et al., 1995; Dupressoir et al., 2001; Mittal et al., 2011; Basquin et al., 2012). The nuclease module appears to contain a single Caf1 and Ccr4 subunit bound to a single Not1 (CNOT1) MIF4G domain. In vertebrate organisms, the presence of two paralogs of Caf1 as well as the occurrence of two highly related Ccr4 subunits thus suggests that the Ccr4-Not complex is heterogeneous. Both human Caf1 paralogs can bind to the human CNOT1 subunit. In addition, both Caf1 proteins can interact with either one of the Ccr4 paralogs and no clear binding preference has been identified (**Figure 3B**).

Other modules of the Ccr4-Not complex include the CNOT11/CNOT10 module, which is attached to the amino-terminus of CNOT1 (Not1) that largely contains α-helical HEAT repeats (Basquin et al., 2012; Bawankar et al., 2013; Mauxion et al., 2013). A central region encompassing a DUF3819 domain interacts with the RQCD1/RCD1/Caf40/CNOT9 subunit, which is composed of Armadillo repeats forming a bundle of α-helices (Garces et al., 2007; Bawankar et al., 2013). This subunit has the ability to bind single and double stranded nucleic acids (Garces et al., 2007). Interestingly, the affinity for sequences containing G/C/T is much greater than oligo(A). The carboxy-terminus of CNOT1 is bound by CNOT2 (Not2), which interacts with CNOT3 (Not3/Not5) via their carboxy-terminal Not-Box region (Bai et al., 1999; Bawankar et al., 2013; Bhaskar et al., 2013; Boland et al., 2013). In yeast, this region also interacts with the Not4 ubiquitin protein ligase subunit (Bai et al., 1999). However, in human cells, the homologs CNOT4 subunit does not bind stably to the CNOT1 protein (Lau et al., 2009).

In addition to the nuclease subunits, several non-catalytic subunits are implicated in mRNA deadenylation (Tucker et al., 2002; Temme et al., 2004; Ito et al., 2011a,b; Takahashi et al., 2012). However, genetic analysis in *Saccharomyces cerevisiae* has shown that the Not-module has additional roles as compared to the Caf1 and Ccr4 subunits (Bai et al., 1999). The additional role of the Ccr4-Not complex is less well defined as compared to its function in deadenylation, and may include transcriptional regulation, and/or an involvement in the co-translational control of protein folding (Collart and Panasenko, 2012).

## SPECIALIZED OR REDUNDANT ROLES FOR THE Caf1 and Ccr4 PARALOGS?

**FIGURE 4:**
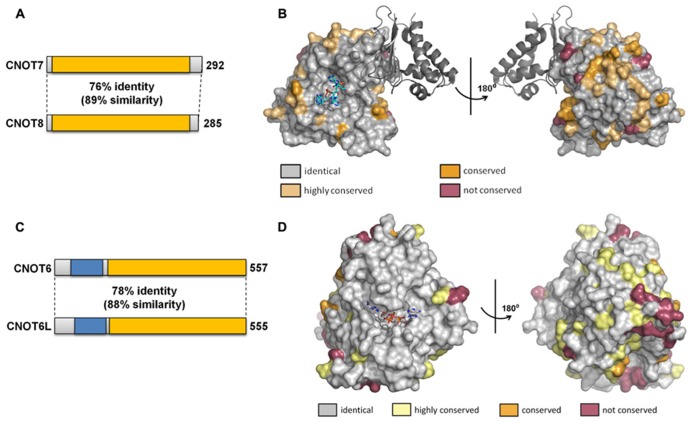
**Similarity of the Caf1 and Ccr4 paralogs.** **(A)** Schematic overview of similarity between the Caf1 paralogs encoded by *CNOT7* and *CNOT8*. The conserved DEDD nuclease domain is indicated in yellow. **(B)** Conservation of surface residues between the Caf1/CNOT7 and Caf1/CNOT8 subunits. The surface of the human Caf1/CNOT7 protein is shown with residues identical in Caf1/CNOT7 and Caf1/CNOT8 shown in gray, conserved residues in tan or dark yellow, and non-conserved residues in purple. The BTG domain of TOB1 is shown in dark gray. The image was generated using PDB file 2D5R. **(C)** Schematic overview of similarity between the Ccr4 paralogs encoded by *CNOT6* and *CNOT6L*. The conserved EEP nuclease domain is indicated in yellow, the LRR domain in blue. **(D)** Conservation of surface residues between the Ccr4/CNOT6 and Ccr4/CNOT6L subunits. The surface of the human Ccr4/CNOT6L protein is shown with residues identical in Ccr4/CNOT6 and Ccr4/CNOT6L shown in gray, conserved residues in tan or dark yellow, and non-conserved residues in purple. The image was generated using PDB file 3NGO.

The duplication of the genes encoding the Caf1 and Ccr4 subunits in vertebrate cells led to the suggestion that the paralogs might have slightly specialized roles in mRNA deadenylation. So far, this question has been addressed in most detail by studying the human paralogs. The human Caf1 proteins (76% identity, 89% similarity at the amino acid level) are encoded by the *CNOT7* and *CNOT8* genes (**Figure 4A**). The surface residues in the active site are completely conserved (**Figure 4B**). Despite the high sequence conservation, however, the enzymatic activity of the purified Caf1/CNOT7 and Caf1/CNOT8 proteins differs substantially. Whereas both proteins display selectivity toward poly(A), the Caf1/CNOT8 protein appeared to have a significantly higher turnover rate (Bianchin et al., 2005). By contrast, following a systematic approach to identify interacting proteins of Ccr4-Not subunits, a more limited role was proposed for the Caf1/CNOT8 subunit as compared to Caf1/CNOT7. In this study, proteins involved in splicing were not found to associate with Caf1/CNOT8, and a less stable association with Ccr4/CNOT6 was proposed (Lau et al., 2009). However, the role of Caf1/CNOT7 and Caf1/CNOT8 in the regulation of mRNA levels is essentially identical when studied in human breast cancer cells using knockdown approaches in combination with genome-wide expression profiling (Aslam et al., 2009). Upon knockdown of either CNOT7 or CNOT8 mRNA, modest changes were observed on a limited set of mRNAs. By contrast, quantitatively significant effects were observed upon combined knockdown of CNOT7 and CNOT8. Thus, from this study it appears that the Caf1/CNOT7 and Caf1/CNOT8 paralogs do not have intrinsically different properties in the regulation of mRNA levels in cells.

The human Ccr4 proteins (78% identity, 88% similarity at the amino acid level) are encoded by the *CNOT6* and *CNOT6L* genes (**Figure 4C**). As is the case for the human Caf1 paralogs, the surface residues in the active site are completely conserved in Ccr4/CNOT6 and Ccr4/CNOT6L (**Figure 4D**). In cells, these paralogs appear to have essentially identical properties as determined using a knockdown approach in combination with microarray-based expression profiling (Mittal et al., 2011). Whereas CNOT6 knockdown has little effect on mRNA levels as compared to CNOT6L knockdown, the combined knockdown of CNOT6 and CNOT6L quantitatively changes the differential expression pattern observed upon CNOT6L knockdown.

The importance of duplications of the genes encoding Caf1 and Ccr4 remains unclear. It may be speculated that, whilst the intrinsic properties of the Caf1 paralogs on the one hand, and of the Ccr4 paralogs on the other, appear to be essentially identical, duplication of the genes encoding the Caf1 and Ccr4 subunits may have allowed fine-tuning of tissue-specific expression of these proteins in vertebrata. Therefore, whilst the *CNOT7* and *CNOT8* genes, as well as *CNOT6* and *CNOT6L*, respectively, may be largely redundant, they may have cell-type specific functions. For instance, Ccr4/CNOT6L, but not Ccr4/CNOT6, is required for proliferation of mouse 3T3 fibroblast cells (Morita et al., 2007). By contrast, both Ccr4/CNOT6 and Ccr4/CNOT6L are required for proliferation of human MCF-7 breast carcinoma cells (Mittal et al., 2011). Moreover, while both Ccr4 paralogs are involved in the maintenance of MCF-7 cell viability, no such role has been documented for Ccr4/CNOT6L in mouse 3T3 cells (Morita et al., 2007; Mittal et al., 2011).

## THE BTG/TOB PROTEINS

The BTG/TOB proteins are the best characterized interaction partners of the Caf1 proteins in vertebrates (Mauxion et al., 2009; Winkler, 2010). These proteins interact with the Caf1 protein via their conserved amino-terminal BTG domain, which interacts at a site away from the Caf1 active site residues (**Figure 3C**; Horiuchi et al., 2009). When over-expressed, these proteins inhibit cell cycle progression, which is dependent on interactions with the Caf1 proteins (Horiuchi et al., 2009; Doidge et al., 2012; Ezzeddine et al., 2012). Interestingly, the number of these proteins appears to have expanded during evolution. Single cellular eukaryotes do not contain a gene encoding a BTG/TOB protein, but BTG/TOB proteins are present in *Caenorhabditis elegans* (1), *Drosophila melanogaster* (2), mouse (6), and human (6) cells. In their carboxy-termini, the TOB1 and TOB2 proteins are highly similar and contain a conserved PAM2 motif that is able to interact with the PABP C-terminal domain (Ezzeddine et al., 2007). Similarly, the BTG1 and BTG2 proteins, which contain a short carboxy-terminal extension, are highly related. Thus, an intriguing number of possible interactions may occur between TOB1/TOB2 or BTG1/BTG2 and the Caf1 paralogs in vertebrate cells. Although the use of genetically engineered mice lacking the Tob1, Tob2, Btg1, or Cnot7 gene have started to uncover the significance of specific roles of the paralog genes, the fundamental importance of this network remains presently unclear (Yoshida et al., 2000; Nakamura et al., 2004; Park et al., 2004; Ajima et al., 2008).

*In vitro*, BTG2 can inhibit the activity of purified Caf1, although the conserved BTG domain of TOB1 does not affect the activity of Caf1 (Yang et al., 2008; Horiuchi et al., 2009). In addition, it was reported that TOB1 can inhibit the deadenylase activity of immunopurified GFP-CNOT6L (Miyasaka et al., 2008). However, functional studies based on reporter genes indicate that BTG2, TOB1, and TOB2 are factors promoting deadenylation and mRNA degradation in cellular transfection assays (Ezzeddine et al., 2007; Mauxion et al., 2008; Doidge et al., 2012; Ezzeddine et al., 2012).

## COLLABORATION OR SPECIALIZATION OF Caf1 AND Ccr4 SUBUNITS?

At first glance, several findings suggest a collaborative mode of action for Caf1 and Ccr4: firstly, both proteins have a preference for poly(A), and, secondly, the subunits directly interact. However, as explained above, it is ambiguous whether the *Saccharomyces cerevisiae* Caf1 is an active deadenylase *in vivo*, indicating that the yeast Ccr4 subunit may be the only active catalytic subunit (Tucker et al., 2002; Thore et al., 2003; Viswanathan et al., 2004). Moreover, the (partial) crystal structure of the yeast Not1-Caf1-Ccr4 sub-complex shows that the catalytic centers are distant and pointing in different directions (**Figure 3C**; Basquin et al., 2012). Functional analysis of Caf1 and Ccr4 in human cells also indicate that the proteins do not have identical roles. Comparison of the expression profile of Caf1 knockdown cells with that of Ccr4 knockdown cells shows qualitative differences in the differentially regulated mRNA sets (Mittal et al., 2011). Moreover, the phenotypes of Caf1 knockdown cells are different to that of Ccr4 knockdown cells. Whereas cell proliferation is reduced upon knockdown of either Caf1 or Ccr4, Ccr4 is required for cell viability and the prevention of cellular senescence. Thus, these findings suggest that the Caf1 and Ccr4 subunits may have specialized roles.

It is unclear what the mechanistic basis is for the differential role in mRNA deadenylation of the Caf1 and Ccr4 subunits. It may be speculated that the Ccr4-Not complex may be distinctly arranged depending on the mechanism of recruitment by protein–protein interactions. Thus, depending on the particular proteins involved, either the Caf1 or the Ccr4 subunit may be placed in a suitable orientation for deadenylation.

## COOPERATION AND REDUNDANCY WITH OTHER DEADENYLASE COMPLEXES

Whilst the Ccr4-Not complex is described as the main deadenylase in a variety of organisms, including *Saccharomyces cerevisiae* and human cells (Tucker et al., 2001; Yamashita et al., 2005), it is clear that other deadenylase complexes, notably the Pan2-Pan3 complex, also play an important role. Using transcriptional pulse experiments of reporter mRNAs in human cells, it has been established that Pan2-Pan3 is important for the early stage of deadenylation in human cells, with the Ccr4-Not components involved in a distinct, second stage (Yamashita et al., 2005; Zheng et al., 2008). Interestingly, interactions between Pan2-Pan3 and Ccr4-Not can be detected suggesting that both deadenylase complexes can reside in a higher order complex that may include other components (Zheng et al., 2008). Thus, these experiments suggest that Pan2-Pan3 and Ccr4-Not may have unique, but cooperative roles, in mRNA decay. On the other hand, genetic analyses in the budding yeast *Saccharomyces cerevisiae* indicate that yeast *pan2Δ* or *ccr4Δ* cells lacking a single catalytic deadenylase subunit are viable and do not display significant growth phenotypes (Boeck et al., 1996; Brown et al., 1996; Tucker et al., 2001). By contrast, yeast *pan2Δ* *ccr4Δ* cells lacking both catalytic subunits display a strong synthetic phenotype indicating that the Pan2-Pan3 and Ccr4-Not complexes are largely redundant and can compensate for each other’s loss of function (Boeck et al., 1996; Brown et al., 1996; Tucker et al., 2001).

## PERSPECTIVE AND CONCLUDING REMARKS

Recently, significant progress has been made to understand the importance of deadenylation by the Ccr4-Not complex. Despite this, fundamental questions remain regarding the significance of the duplications of genes encoding the Ccr4-Not nuclease subunits, and the enzymatic mechanism of deadenylation by Ccr4-Not. The functional significance of paralogs encoding the nuclease subunits and of the (potential) redundancy with other deadenylases may be addressed by using mouse knockout models and mice engineered to have point mutations resulting in catalytically inactive deadenylase subunits. Such experiments may be facilitated by advances in genome engineering tools, such as the use of zinc finger nucleases or RNA-guided genome editing using the CRISPR/Cas system. Additionally, the use of novel approaches, such as the use of chemical probes of deadenylase enzymes that specifically inhibit their enzymatic activity without affecting structural roles, should be explored to further the understanding of deadenylation by the Ccr4-Not complex (Maryati et al., 2013).

## Conflict of Interest Statement

The authors declare that the research was conducted in the absence of any commercial or financial relationships that could be construed as a potential conflict of interest.
